# Late Toxicity and Long‐Term Quality of Life in Survivors of Cancer of the Major Salivary Glands More Than 5 Years After Diagnosis: A Multi‐National Study

**DOI:** 10.1002/hed.28263

**Published:** 2025-08-01

**Authors:** Susanne Singer, Cecilie Delphin Amdal, Kristin Bjordal, Bente Brokstad Herlofson, Guro Lindviksmoen Astrup, Andreas Boehm, Ulrike Wöhner, Eva Hammerlid, Ricardo Ribeiro Gama, Alexandre Arthur Jacinto, Femke Jansen, Irma M. Verdonck‐de Leeuw, Tatiana Dragan, Fréderic Duprez, Naomi Kiyota, Monica Pinto, Maximilian Krüger, Orlando Guntinas‐Lichius, Johanna Inhestern, Francesco Tramacere, Pierluigi Bonomo, Giuseppe Fanetti, Sandra Nuyts, Michaela Plath, Noa Stempler, Kristine Løken Westgaard, Katherine J. Taylor

**Affiliations:** ^1^ Department of Quality of Life in Oncology, Comprehensive Cancer Center Mecklenburg‐Vorpommern (CCC‐MV) University Medical Center Rostock Germany; ^2^ Department of Oncology Oslo University Hospital Oslo Norway; ^3^ Department of Research Support Services Oslo University Hospital Oslo Norway; ^4^ Faculty of Medicine University of Oslo Oslo Norway; ^5^ Department of Oral Surgery and Oral Medicine, University of Oslo, and Department of Otorhinolaryngology—Head and Neck Surgery Division for Head, Neck and Reconstructive Surgery Oslo University Hospital Oslo Norway; ^6^ Department of Otolaryngology Head and Neck Surgery St. Georg Hospital Leipzig Germany; ^7^ Department of Otorhinolaryngology‐Head and Neck Surgery Institute of Clinical Sciences, Sahlgrenska Academy at University of Gothenburg, Sahlgrenska University Hospital Gothenburg Sweden; ^8^ Department of Head and Neck Surgery Barretos Cancer Hospital Barretos Brazil; ^9^ Department of Radiation Oncology Barretos Cancer Hospital Barretos Brazil; ^10^ Department Otolaryngology‐Head and Neck Surgery Amsterdam UMC Amsterdam the Netherlands; ^11^ Department of Clinical, Neuro and Developmental Psychology Vrije Universiteit Amsterdam Amsterdam the Netherlands; ^12^ Department of Radiation Oncology, Head and Neck Unit Institut Jules Bordet, Université Libre de Bruxelles Brussels Belgium; ^13^ Department of Radiotherapy‐Oncology Ghent University Hospital, Faculty of Medicine and Health Sciences—Human Structure and Repair, Ghent University Ghent Belgium; ^14^ Department of Medical Oncology and Hematology Kobe University Hospital Cancer Center Kobe Japan; ^15^ Rehabilitation Medicine Unit Istituto Nazionale Tumori—IRCCS—Fondazione G. Pascale Napoli Italy; ^16^ Department of Maxillofacial Surgery University Medical Centre Mainz Germany; ^17^ Department of Otorhinolaryngology Jena University Hospital Jena Germany; ^18^ Department of Otorhinolaryngology Oberhavelkliniken Hennigsdorf Hennigsdorf Germany; ^19^ Department of Radiation Oncology Azienda Sanitaria Locale Brindisi Italy; ^20^ Radiation Oncology Azienda Ospedaliero‐Universitaria Careggi Florence Italy; ^21^ Division of Radiation Oncology Centro di Riferimento Oncologico di Aviano (CRO) IRCCS Aviano Italy; ^22^ Department of Radiation Oncology, Leuven Cancer Institute University Hospitals Leuven, Laboratory of Experimental Radiotherapy, Department of Oncology Leuven Belgium; ^23^ Department of Otorhinolaryngology, Head and Neck Surgery University Hospital Heidelberg Heidelberg Germany; ^24^ Oral Medicine Unit Sheba Medical Center Tel Hashomer Israel; ^25^ Division of Epidemiology and Health Services Research, Institute of Medical Biostatistics, Epidemiology, and Informatics (IMBEI) University Medical Centre of Johannes Gutenberg University Mainz Germany

**Keywords:** EORTC QLQ‐C30, EORTC QLQ‐HN35, late toxicity, long‐term quality of life, patient‐reported outcomes

## Abstract

**Background:**

Because salivary gland cancers (SGC) are rare and include different tumor subtypes, data on their long‐term quality of life and late toxicities are sparse.

**Methods:**

Multi‐national study including SGC survivors more than 5 years after diagnosis. They completed the European Organisation for Research and Treatment of Cancer (EORTC) quality of life core questionnaire together with its head and neck cancer module and reported problems that were most bothering for them. Toxicity was clinically assessed.

**Results:**

Altogether, 60 survivors from nine countries participated and reported *dry mouth* (mean score 37.2), *use of painkillers* (35.0), *problems with sexuality* (30.1), *insomnia* (28.9), *fatigue* (27.8), *trismus* (24.9), and *sticky saliva* (23.3). The most frequently mentioned long‐term problem was *dry mouth*, mentioned by a third of all participants. The most frequent toxicities were *hearing impairment*, *soft tissue fibrosis*, *dry mouth*, and *cranial neuropathies*.

**Conclusions:**

Dry mouth is a frequent and disturbing problem in SGC survivors.

## 1 Introduction

Salivary gland cancers are a group of malignant tumors in the parotid, submandibular, sublingual glands, or, rarely, in the minor salivary glands of the head and neck area. It is a rare condition, with an estimated number of cases (1‐year prevalence) of about 42 000 worldwide (Globocan 2022; https://gco.iarc.who.int). About 24% of the patients live in Eastern Asia, followed by South Central Asia (17%), Northern America (13%), and South‐Eastern Asia (9%). The age‐standardized incidence worldwide is 0.57 per 100 000 inhabitants, ranging from 0.07 in the Republic of the Gambia to 1.46 in Togo. Men are diagnosed more frequently than women (0.67 vs. 0.48). About 24 000 people die each year from salivary gland cancers; the highest mortality in 2022 was found on the Solomon Islands (1.15), the lowest in Puerto Rico and Luxembourg (both 0.04).

The major salivary glands have different proportions of secretory acini, affecting the saliva composition [1]. The parotid gland produces serous saliva, which is a watery, enzyme‐rich secretion that aids in carbohydrate digestion. The submandibular and sublingual glands contribute to saliva production, especially during unstimulated states. They produce mixed serous and mucous saliva, providing both enzymatic and lubricating properties [1]. Bilateral synchronous or metachronous salivary gland cancer is very rare; only a few case reports are published [2, 3, 4, 5].

There are more than 20 different types of malignant tumor histopathology, with the treatment depending on both cancer stage and histopathology. Smaller tumors can be treated with local surgery only, provided it is not an aggressive cancer, while larger tumors require a combination of surgery and radiation. After surgical resection of a salivary gland tumor, radiotherapy is indicated only for malignant tumors with high‐risk features such as positive margins, high‐grade tumors, or lymph node involvement. Both chemotherapy and targeted therapy alone or in combination with local treatment are administered only in very advanced or metastatic disease [6, 7]. It is therefore usually not received by long‐term survivors. Hormonal therapy can be chosen as a systemic option in androgen‐receptor positive salivary gland cancer [7].

Because salivary gland cancers are rare and include such a large variety of different tumor subtypes, data on their (long‐term) health‐related quality of life and on late toxicities are sparse. Examples of known problems related to salivary gland tumor treatment include facial nerve paralysis [8], with resulting functional problems (dysphagia, difficulties with articulating, facial disfigurement, difficulties with eating, keratopathy—which can eventually lead to blindness [9]—and nasal obstruction due to nasal alar collapse), hypo‐ and hyperesthesia, pain, dry mouth, dry eyes, swallowing problems, and fear of recurring disease [10, 11]. The latter is especially true for patients with adenoid‐cystic carcinoma; their risk of distant metastasis (primarily to the lungs) is high even after 20 years, so these patients may need life‐long follow‐up, much longer than is usual for head and neck cancer. A parotidectomy can also lead to Frey's syndrome [12], where patients suffer from increased sweating after gustatory stimuli. It has been reported in up to 69% of all cases, depending on the extent of the surgery [13]. A parotidectomy can also lead to first bite syndrome, which causes pain when salivating or taking the first bites, which appears to affect quality of life negatively [14, 15]. The incidence has been reported to be about 10%, but with varying numbers depending on the extent of surgery [15].

Another problem frequently reported by patients after parotidectomy is hypoesthesia of the skin, even up to 2 years after the surgery [16], but there is conflicting evidence as to what extent this is disturbing for patients [14, 16, 17, 18].

Swallowing problems and dry mouth, in contrast, are clearly rated as very disturbing both by patients during and after treatment as well as by long‐term survivors [19, 20, 21]. There are not many studies on salivary flow dynamics after salivary gland surgery. After parotidectomy for benign tumors, there seems to be no change after surgery due to compensatory mechanisms of the contralateral gland [22, 23, 24]. Not much is known about these effects in salivary gland cancer. If the patients received surgery to only one gland, this usually is not a significant problem, but if they receive radiotherapy to the other remaining salivary glands, it can have a notable impact on saliva production [25]. This, however, is rarely needed.

In summary, salivary gland cancers are heterogeneous in terms of histology, treatment pathways, and prognosis. This heterogeneity, together with the rarity of the disease as a whole, results in few data published on patients' and especially on long‐term survivors' health‐related quality of life and late toxicities. Only by combining data from multiple sites can sufficient numbers for reliable conclusions be drawn. With our international study, we aimed to better understand the extent of health‐related quality of life constraints and toxicities in salivary gland cancer survivors in the long term.

## 2 Methods

### 2.1 Study Design

We conducted a multi‐centre cross‐sectional study (“Late Toxicity and Long‐term Quality of Life in Head and Neck Cancer Survivors”, EORTC 1629). The methods have been described in detail elsewhere [26]. In brief: Members of the EORTC Quality of Life Group and the EORTC Head and Neck cancer group were invited to participate in this study. Those who agreed (“collaborators” in the following) were asked to identify head and neck cancer patients from their records and invite them via e‐mail, phone, or letter to come to the hospital for a clinical check‐up. A doctor, not necessarily the one who had treated the patients originally, met the survivors in person and asked them about potential late effects. They also performed clinical tests to be able to judge whether a toxicity was present or not. The patients also completed a set of validated questionnaires ascertaining their health‐related quality of life (see below for details).

Written informed consent was obtained from each participant. Ethical approval was granted from the responsible board of the principal investigator (Landesärztekammer Rheinland‐Pfalz, No. 2018‐13579), and it was obtained at each site according to the local regulations.

### 2.2 Inclusion and Exclusion Criteria

Patients were eligible for this analysis if they had been diagnosed with cancer of the major salivary glands (ICD‐10 code C07 or C08) more than 5 years before (no upper limit of time since diagnosis), aged 18 years or older at the time of study enrollment and able to participate in the clinical examination. All other cancer sites were excluded from this analysis. The collaborators were asked to contact all of their patients who were eligible. They should not select certain patients in order to reduce selection bias.

### 2.3 Instruments

Health‐related quality of life was measured using two instruments developed by the European Organisation for Research and Treatment of Cancer (EORTC): the core questionnaire (EORTC QLQ‐C30) and the corresponding module for head and neck cancer (EORTC QLQ‐H&N35).

The EORTC QLQ‐C30 contains 30 items asking about different domains of quality of life that are often impaired because of cancer and its treatment [27]. The EORTC QLQ‐H&N35 addresses quality of life issues specifically relevant for patients with malignant tumors of the head and neck [28]. We decided to use the original version of the questionnaire rather than the updated one [29] because the patients had been treated a long time ago, and the older version of the module was considered to be more in line with the treatment procedures at that time.

The patients were also asked by the clinicians to name their top two most serious late effects. They could choose from a list presented to them but could also mention other late effects which were then documented in writing.

The participants also completed a questionnaire based on the Treatment Inventory of Costs in Patients (TiCP) [30], which includes questions about how frequently health care professionals were visited during the past 3 months. We expanded this time frame to 12 months and included a question on visits to a dentist.

Toxicity was documented according to the Common Terminology Criteria for Adverse Events (CTCAE), version 5. The CTCAE is a terminology system for toxicities, where the severity of a given adverse event can be graded from 1 (asymptomatic or mild symptoms) to 5 (death related to the adverse event). Based on the literature and our clinical experience, we focused our analysis on the following toxicities: dry mouth, dysphagia, soft tissue fibrosis (neck fibrosis), trismus, oral pain, neck pain, pharyngolaryngeal pain, face edema, neck edema, osteoradionecrosis, hearing impairments, olfactory nerve disorder, facial nerve disorder, acoustic nerve disorder, and accessory nerve disorder.

In some sites, where a dentist was available, observer‐rated oral dryness was assessed using the Clinical Oral Dryness Score (CODS) [31]. The CODS assesses dry mouth based on a clinical and visual examination of the oral cavity using various signs of dry mouth, such as foamy saliva, friction of the oral mucous membranes, and stickiness of the teeth on the tongue or cheek folds. It has been shown to be associated with unstimulated and stimulated salivary flow in patients with hyposalivation [32].

The clinicians documented the type of anti‐cancer treatment received, the clinical characteristics (ICD code, histology, cancer stage according to the Union Internationale Contre le Cancer [UICC], current evidence of disease, Charlson comorbidity index [33]) and demographic characteristics (gender, current age, cohabitation, education).

## 3 Data Analysis

We defined a priori the following quality of life domains as being of primary interest: *pain in the mouth, swallowing problems, problems with senses, problems with social eating, problems with social contact, problems with sexuality*, *problems with teeth, problems opening mouth, dry mouth, sticky saliva, emotional functioning*, and *global quality of life*.

For these domains, we compared the scores between patients who had received **s**urgery alone vs. surgery plus radiotherapy. These group comparisons were done with linear regression, while adjusting for UICC stage and age. For comparison, we computed the scores of other head and neck cancer survivors from our study who also had received multimodal treatment. We also calculated the scores of survivors who had received 3D conformal radiotherapy and those with intensity‐modulated radiation therapy (IMRT), of men and women, and of patients with parotid tumors vs. other tumors separately. For the toxicities, we calculated the proportion of each severity grade.

For the top two most serious long‐term effects from the perspective of the patients, we read their answers, coded them, and counted the codes, all using a software for qualitative data analysis, MAXQDA (*VERBI Software*, Consult Sozialforschung GmbH, Berlin, Germany). Quantitative analysis was performed using STATA (StataCorp. 2017. *Stata Statistical Software: Release 15*. College Station, TX: StataCorp LLC).

## 4 Results

### 4.1 Sample

In this study, 60 long‐term survivors of salivary gland cancer from 9 countries (Belgium *n* = 7, Brazil *n* = 5, Germany *n* = 12, Israel *n* = 1, Italy *n* = 6, Japan *n* = 2, The Netherlands *n* = 5, Norway *n* = 17, Sweden *n* = 5) participated. Survivor enrollment took place between 2018 and 2021. The time since diagnosis ranged from a bit more than 5 to 21 years; on average, it was 10 years (years of diagnosis: 1998 to 2016). Four survivors had evidence of disease at the time of the study, either due to a recurrence or a second primary; 11 participants had experienced a recurrence at some point since their diagnosis. The participants were on average 64 years old at the time of the study; the youngest was 23 years and the oldest 90 years old. More characteristics of the participants are displayed in Table 1.

**TABLE 1:** Patient characteristics (*n* = 60).

Variable	Category	Number	Percent
Gender	Male	31	52%
	Female	29	48%
Education at school	< 10 years	14	23%
	10 years	7	12%
	≥ 10 years	37	62%
	Unknown	2	3%
Cohabitation	Lives alone	9	15%
	Lives with partner +/− children	47	78%
	Lives with other people	4	7%
Subsite	Parotid gland (ICD 10, C07)	47	78%
	Other salivary gland (ICD 10, C08)	13	22%
UICC (version 7)	I	20	33%
	II	16	27%
	III	11	18%
	IV	11	18%
	Unknown	2	3%
Histology	Mucoepidermoid carcinoma	11	18%
	Adenoid cystic carcinoma	11	17%
	Acinic cell carcinoma	9	15%
	Adenocarcinoma	7	12%
	Squamous cell carcinoma	7	12%
	Salivary duct carcinoma	5	8%
	Myoepithelial carcinoma	5	8%
	Sarcoma	1	2%
	Epithelial‐myoepithelial carcinoma	1	2%
	Pleomorphic adenoma	1	2%
	Neuroendocrine carcinoma	1	2%
	Carcinosarcoma	1	2%
History	Recurrence (ever since first diagnosis)	11	18%
	Patient has had a second primary before or after the salivary gland cancer diagnosis	9	15%
	… prostate cancer	3	33%
	… other head and neck cancer	2	22%
	… lung cancer	2	22%
	… breast cancer	1	11%
	… thyroid cancer	1	11%
Treatment	Surgery only	11	18%
	Surgery with radiotherapy	49	82%
	… with 3D conformal radiotherapy	26	43%
	… with IMRT	21	35%
	… with IMRT and cyberknife	1	2%
	… unknown type of radiotherapy	1	2%
Type of surgery	Transoral/transnasal/endoscopic without neck dissection	5	8%
	Transoral/transnasal/endoscopic with neck dissection	11	18%
	Transcervical (with or without neck dissection)	34	57%
	Neck dissection only	1	2%
	Other	9	15%
Smoking status	Never smoker	35	58%
	Former smoker	23	38%
	Current smoker	1	2%
	Unknown	1	2%
Alcohol consumption	Never	16	27%
	Monthly or less	7	12%
	2 to 4 times a month	14	23%
	2 to 3 times a week	12	20%
	4 to 5 times a week	9	15%
	Unknown	2	3%
Performance score	KPS = 60	1	2%
	KPS = 70	3	5%
	KPS = 80	17	28%
	KPS = 90	12	20%
	KPS = 100	25	42%
Comorbidity	CCI = 0	37	62%
	CCI = 1	9	15%
	CCI > 1	14	23%

Abbreviations: CCI, Charlson Comorbidity Index; KPS, Karnofsky Performance Score; UICC, Union Internationale Contre le Cancer.

### 4.2 Health‐Related Quality of Life

**FIGURE 1:**
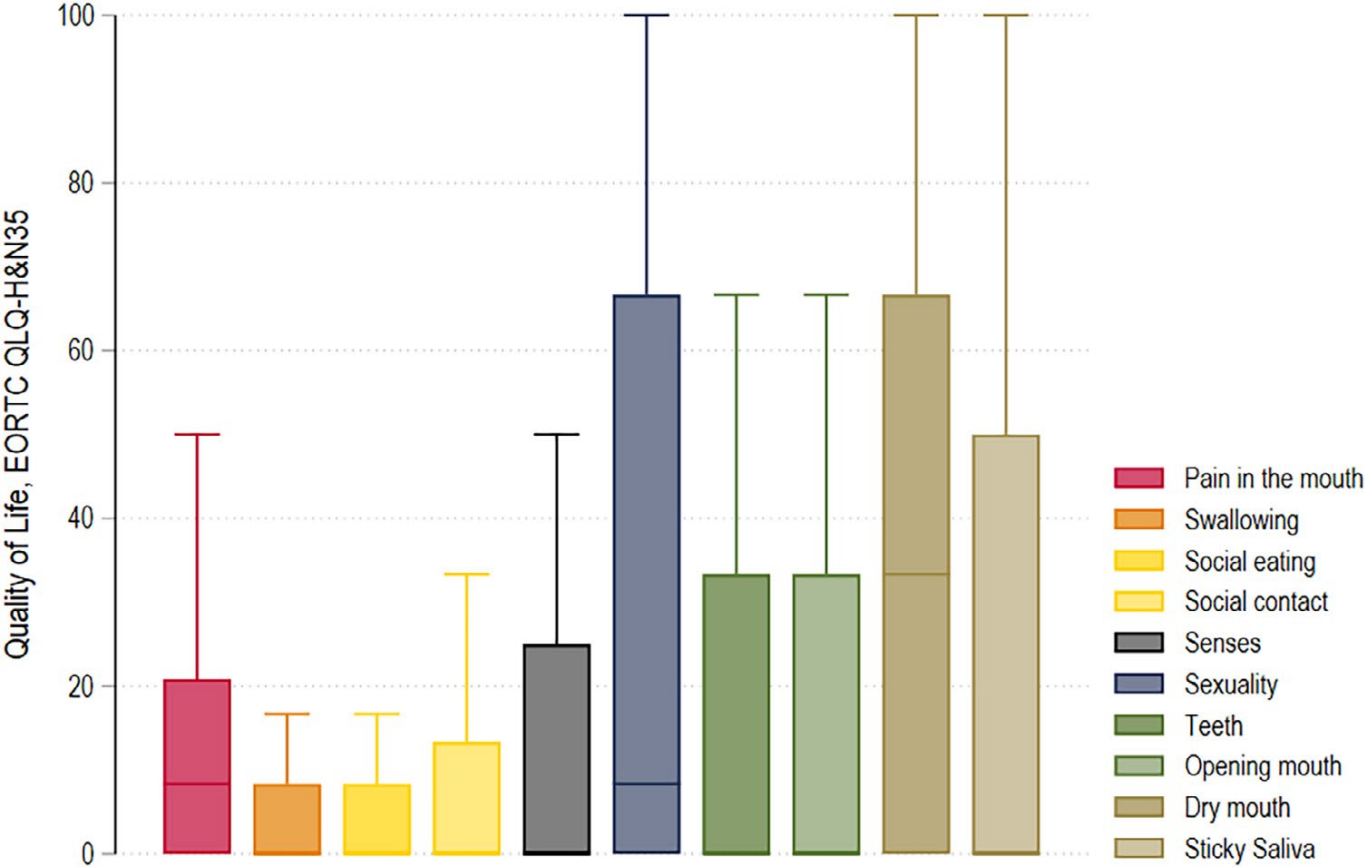
Head and neck cancer specific quality of life in salivary gland cancer survivors. Displayed are the medians and interquartile ranges per scale. High scores indicate high symptom burden. Abbreviations: B, regression coefficient; CI, confidence interval. [Color figure can be viewed at wileyonlinelibrary.com]

The highest scores, indicating high symptom burden, were reported for the following domains: *dry mouth* (mean score 37.2), *use of painkillers* (35.0), *problems with sexuality* (30.1), *insomnia* (28.9), *fatigue* (27.8), *problems opening mouth* (24.9), and *sticky saliva* (23.3) (Figure 1 and Table 2).

**TABLE 2:** Health‐related quality of life of salivary cancer survivors.

Area	Domain	Mean	SD	Minimum	Maximum
General quality of life (EORTC QLQ‐C30)	# Physical functioning	84.3	18.1	33	100
	# Role functioning	81.4	28.6	0	100
	# Emotional functioning	79.5	21.2	0	100
	# Cognitive functioning	78.1	25.2	0	100
	# Social functioning	83.9	25.5	0	100
	# Global health status/QoL	70.6	23.5	17	100
	Fatigue	27.8	27.9	0	100
	Nausea and vomiting	2.2	6.5	0	33
	Pain	18.1	27.0	0	100
	Dyspnoea	18.3	27.0	0	100
	Insomnia	28.9	31.6	0	100
	Appetite loss	7.2	19.5	0	67
	Constipation	13.9	27.0	0	100
	Diarrhea	6.7	14.8	0	67
	Financial difficulties	9.4	23.0	0	100
Head and neck specific quality of life(EORTC QLQ‐H&N35)	Pain in the mouth	12.5	15.5	0	58
	Swallowing problems	6.7	10.7	0	50
	Problems with senses	14.4	24.4	0	100
	Problems with speech	10.6	15.6	0	67
	Problems with social eating	6.7	13.3	0	50
	Problems with social contact	8.3	13.5	0	60
	Problems with sexuality	30.1	36.2	0	100
	Problems with teeth	19.4	27.6	0	100
	Problems opening mouth	24.9	30.1	0	100
	Dry mouth	37.2	34.8	0	100
	Sticky saliva	23.3	34.3	0	100
	Coughing	17.2	27.1	0	100
	Feeling ill	16.1	27.8	0	100
	Painkillers	35.0	48.1	0	100
	Nutritional supplements	11.7	32.4	0	100
	Feeding tube	1.7	12.9	0	100
	Weight loss	8.5	28.1	0	100
	Weight gain	33.3	47.5	0	100

*Note*: # denotes Functioning Scales, where high scores indicate good health‐related quality of life. The remaining scales are Symptom Scales, where high scores indicate high symptom burden.

Abbreviation: SD, standard deviation.

**FIGURE 2:**
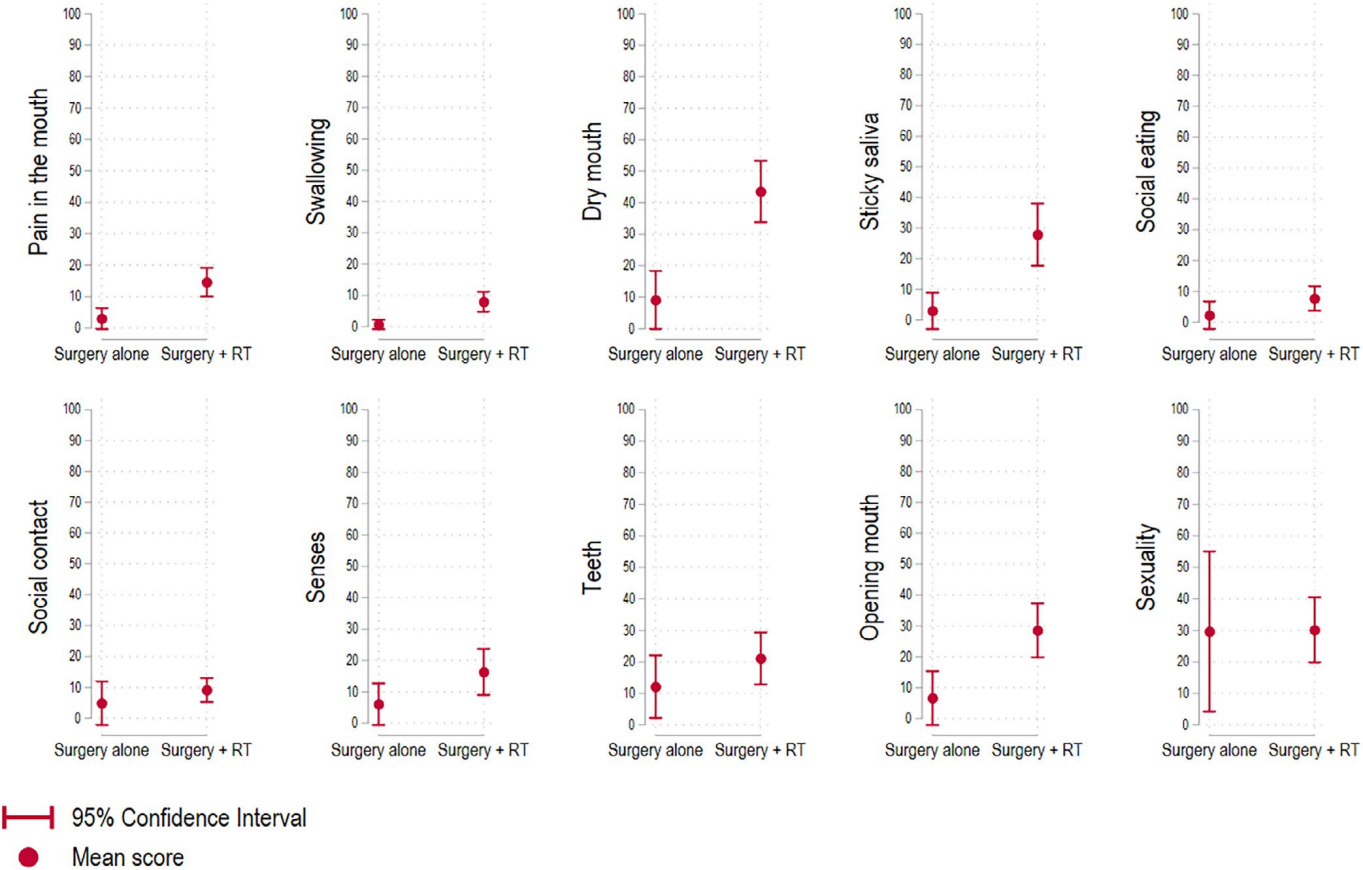
Head and neck cancer specific quality of life by treatment. Displayed are the unadjusted mean scores. High scores represent poor quality of life. Whiskers represent 95% confidence intervals. RT: Radiotherapy. [Color figure can be viewed at wileyonlinelibrary.com]

Survivors who had received multimodal treatment reported more problems with *dry mouth*, *sticky saliva, problems opening mouth*, and *swallowing problems* when adjusting for age and UICC stage (Figure 2 and Table 3). The scores were comparable with other head and neck cancer survivors who had received multimodal treatment (Table 3).

**TABLE 3:** Health‐related quality of life of salivary cancer survivors, by treatment.

	All	Surgery	Surgery +radiotherapy	Other head and neck cancer survivors after multimodal treatment	Other salivary gland cancer patients pre‐treatment	General population	Comparison between patients with and without adjuvant radiotherapy		
Quality of life domain	Mean	Mean	Mean	Mean	Mean	Mean	B	95% CI	*p*
Number of participants	60	11	49	422	225	1504			
Pain in the mouth	12.5	3.0	14.6	17.1	9.5	3.4	10.4	−0.5, 21.2	*0.06*
Swallowing problems	6.7	0.8	8.0	20.6	3.6	1.6	7.9	0.9, 15.0	*0.03*
Dry mouth	37.2	9.1	43.5	47.6	17.5	12.0	38.7	16.4,60.9	*0.001*
Sticky saliva	23.3	3.0	27.9	35.7	7.9	5.9	26.7	3.1, 50.4	*0.03*
Problems with social eating	6.7	2.3	7.7	19.6	6.2	2.6	6.2	−2.9, 15.3	*0.18*
Problems with social contact	8.3	4.8	9.1	11.3	5.1	4.0	2.7	−7.1, 12.4	*0.58*
Problems with Senses	14.4	6.1	16.3	23.7	4.2	4.5	14.9	−2.3, 32.0	*0.09*
Problems with teeth	19.4	12.1	21.1	25.2	11.1	8.8	7.0	−12.6, 26.7	*0.48*
Problems opening mouth	24.9	6.7	28.6	26.8	9.5	2.0	22.1	0.2, 44.0	*0.05*
Problems with sexuality	30.1	29.6	30.1	29.2	9.2	19.2	4.1	−24.1, 32.3	*0.77*
Emotional functioning	79.5	76.5	80.2	78.6	82.1	81.4	5.8	−9.4, 21.0	*0.45*
Global quality of life	70.6	66.7	71.4	69.0	69.8	75.7	4.2	−13.1, 21.4	*0.63*

*Note*: Displayed are the mean scores of quality of life domains for which a difference between patients with and without radiotherapy had been suspected and tested. For reference, data of other head and neck cancer survivors who had received surgery plus radiotherapy [26], of other salivary gland cancer patients before treatment [34], and of the Swedish general population [35] are added. Differences between salivary gland cancer survivors with and without adjuvant radiotherapy were tested with multivariate linear regression adjusted for UICC stage and age.

*Sticky saliva* was numerically more frequent in patients after 3D conformal radiotherapy compared to IMRT (Table S1). Patients with tumors of the parotid glands more often reported *problems with teeth*, with *senses*, and with *sexuality* (Table S2). Women indicated more problems in a variety of quality of life domains, for example, *dry mouth* and *problems opening the mouth* (Table S3).

### 4.3 Most Serious Late Effects From the Perspective of the Patients

**FIGURE 3:**
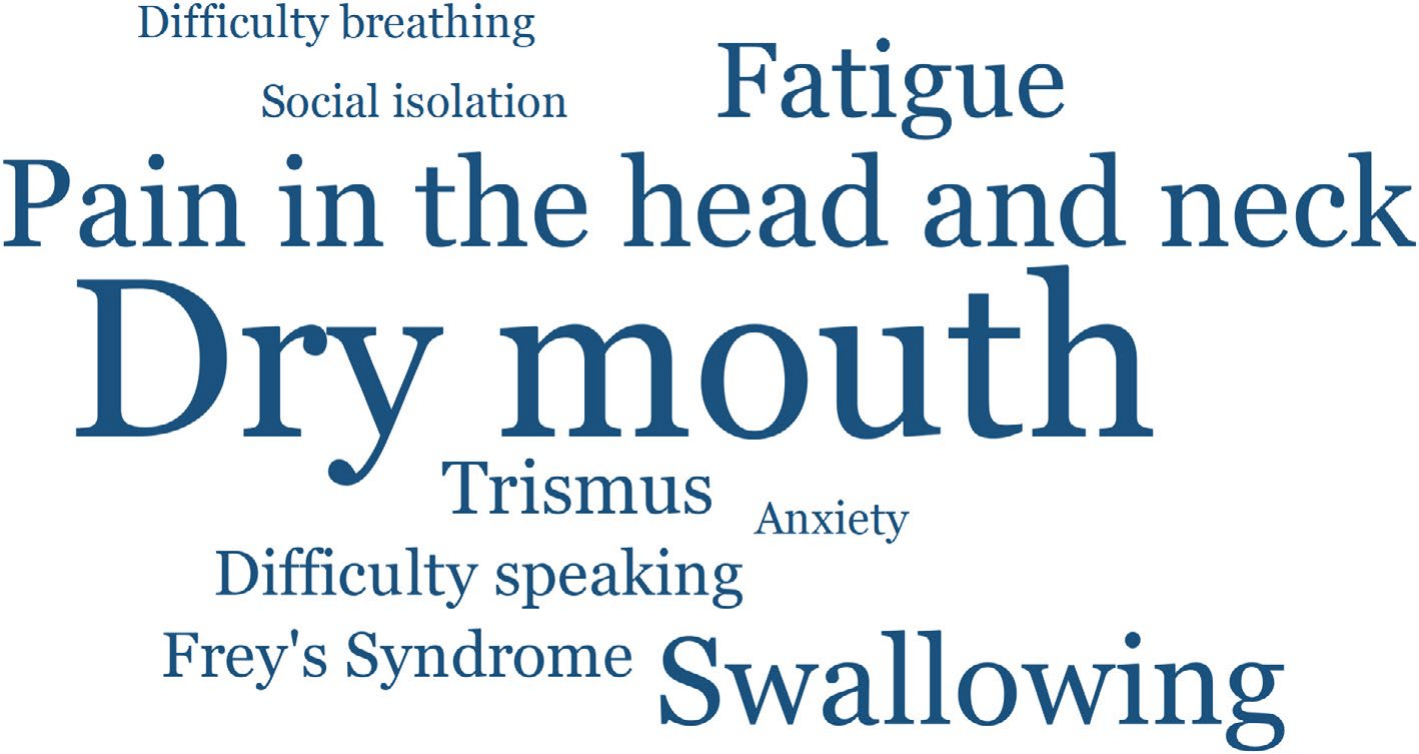
Top long‐term side effects from the perspective of the survivors. The size of the word illustrates the frequency of the mentioning. [Color figure can be viewed at wileyonlinelibrary.com]

Altogether, there were 91 late effects mentioned by the survivors as being the most serious they currently experienced. The most frequently named was *dry mouth*, which was mentioned by a third of all participants (*n* = 21). The second‐most frequent were *pain in the head and neck* as well as *difficulties swallowing*, each mentioned by 10 survivors (Figure 3). This was followed by *fatigue* (*n* = 8), *problems with movement* (*n* = 7) (e.g., moving an arm or having a stiff neck), *emotional problems* (*n* = 6), *trismus* (*n* = 5), *Frey's syndrome* (*n* = 4), *difficulty speaking* (*n* = 4), and *neuropathy* (*n* = 4).

### 4.4 Toxicity

**FIGURE 4:**
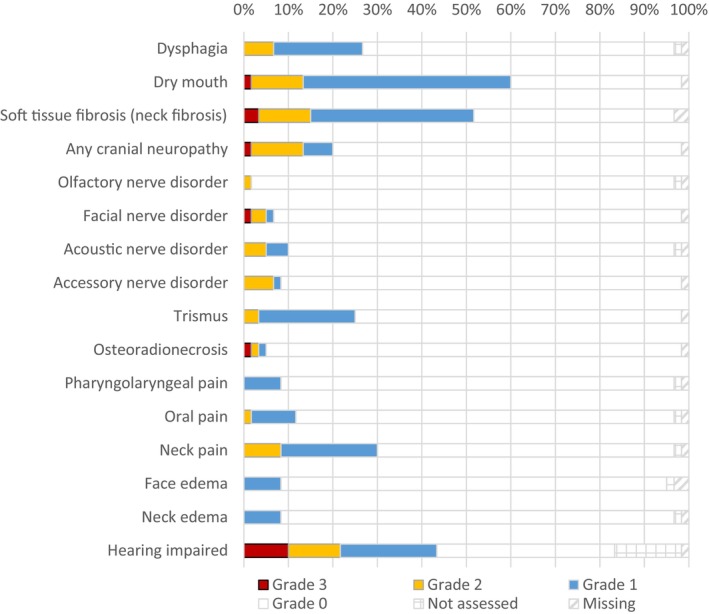
Presence of toxicities in salivary gland cancer survivors as diagnosed by clinicians. [Color figure can be viewed at wileyonlinelibrary.com]

Grade 2 and 3 toxicities combined (no grade 4 toxicities were present) were observed for *hearing impairment* (*n* = 13), *soft tissue fibrosis* (*n* = 9), *dry mouth* (*n* = 8), *cranial neuropathies—any* (*n* = 8), *neck pain* (*n* = 5), *dysphagia* (*n* = 4), *accessory nerve disorder* (*n* = 4), *acoustic nerve disorder* (*n* = 3), *facial nerve disorder* (*n* = 3), *trismus* (*n* = 2), *osteoradionecrosis* (*n* = 2), *olfactory nerve disorder* (*n* = 1), and *oral pain* (*n* = 1). Details are displayed in Figure 4.

Four sites participated in the oral health subproject, and these sites enrolled 24 of the 60 survivors. The clinical assessment of the oral mucosa revealed no abnormalities in 21 of these participants, mucosal atrophy in 2, and lichen planus in 1. The CODS ranged from 0 to 8, with a mean of 2.3 (SD 2.6, median 1). There were no indications of infections or chronic mucositis. The number of teeth lost after treatment (including retained roots treated endodontically due to risk of osteoradionecrosis) ranged from 0 to 4 (mean 0.6, SD 0.9).

### 4.5 Health Care Use

The majority (90%) of the survivors reported having visited a dentist during the past 12 months at least once (Table S1); among these survivors, the number of visits ranged from 1 to 40 in 12 months. General practitioners were the second most commonly visited clinicians (80%), followed by head and neck surgeons/otolaryngologists (37%), cardiologists (13%), and psychotherapists/psychologists/psychiatrists (12%).

Those who visited a dentist reported more severe problems with *dry mouth* (delta: 17) and fewer *problems with opening the mouth* (delta: 22). *Emotional problems* (delta: 16) and *problems with sexuality* (delta: 44) were more severe in survivors who had visited mental health care specialists.

## 5 Discussion

The objective of this study was to better understand potential impairments in quality of life more than 5 years after salivary gland cancer diagnosis as well as the presence of late toxicities in this patient group. We used different methods for this: validated questionnaires, an open‐ended question, and examinations by a clinician.

In all three data sources, dry mouth came out as a frequent and very disturbing problem, especially after postoperative radiotherapy. This issue is known to be of high relevance in the short term [10]. Our study shows that it is also bothersome in the long run and the problems were more common than in another group of salivary gland cancer patients before treatment [34]. Dry mouth is thus often a chronic problem, especially after certain types of radiotherapy—the difference in symptom burden between those who only received surgery versus those with additional radiotherapy is considerable and of clinical importance [36]. Methods to alleviate dry mouth have been investigated but not all are effective. Topical lubricants may be offered to improve dry mouth [37]. Gustatory and masticatory salivary reflex stimulation by sugar‐free lozenges and chewing gum can increase saliva production in people with residual secretory capacity and may be preferred by patients, but there is no evidence that chewing gum is better or worse than saliva substitutes [37]. To date, there is insufficient evidence for the effects of electro‐stimulation devices on dry mouth symptoms [38]. Acupuncture may increase saliva production but the quality of the evidence is poor [38]. It is therefore of utmost importance to prevent dry mouth as much as possible [39]. The patients need regular dental care, especially caries prevention measures, as it is recommended for all cancer patients with persisting xerostomia or in patients with saliva dysfunction like in Sjögren's syndrome [40, 41]. Survivors should be consulted about the possibilities of supportive care options and the importance of oral hygiene, and referred to a specialist if needed. In our study, the majority of the survivors made use of consultations by a dentist or otolaryngologist, especially those with dry mouth.

Swallowing problems appeared not to be of high relevance based on the questionnaire for the majority of the survivors, while for some it was the most serious issue they had. This finding underlines the need to recognize that this group of patients experiences various challenges, and each individual should be asked about their most significant difficulties.

Though pain levels were only moderate to low in the questionnaire, the same instrument showed that the use of painkillers was frequent, which may alleviate the feeling of pain so that the scale score itself was not that high. Pain was one of the most frequently reported problems for the patients when asked directly what bothers them most in the long term. Based on the observer‐rated toxicity assessment, the prevalence of pain was also low, with the highest frequency for neck pain compared to oral pain or pharyngolaryngeal pain. This finding is positive, as head and neck patients in general are known to have a high pain burden in the short term [42].

Fatigue turned out to be a relevant long‐term problem both in the questionnaire and based on the open question. It is an issue some clinicians may avoid asking about because they feel they cannot offer anything to the patients to relieve the suffering (in contrast to pain, for example, where they can prescribe medication) [43, 44]. However, systematic reviews show that exercise and psychosocial counseling may help in reducing and managing cancer‐related fatigue for survivors of other cancers [45, 46]. Whether this is also applicable to salivary gland cancer survivors still needs to be investigated.

Problems with sexuality were reported frequently in the questionnaire but not in response to the clinician asking the open question. This could be due to the fact that some patients may feel shy in talking about this topic. Another explanation could be that it was not included in the list of side effects offered, so the survivors would have to come up with it themselves. Patients with head and neck cancer reported in another study that they preferred the clinician to ask proactively about sexual functioning issues [47]. Our data show that it might be a viable way to ascertain potential problems in a (more anonymous) questionnaire in salivary gland cancer survivors. The high symptom levels, which were considerably higher compared to pre‐treatment data [34], indicate that the problems are common and should not be neglected.

Frey's syndrome was not asked about in the questionnaire and it was not ascertained in a structured way during the clinical examination because it is not a part of the CTCAE, but it was mentioned by four survivors as one of the most serious side effects they suffered from, even though this side effect was not listed, so the survivors had to come up with it themselves. This underlines how important this problem was for them. According to a Cochrane review [48], there is not enough evidence for any specific treatment of this condition, including for botulinum toxin.

The clinical examinations revealed a number of nerve disorders in the long‐term, for example, with regard to the facial nerve, the accessory nerve, and the acoustic nerve. Not much can be done once the nerves are damaged, but it should be kept in mind that these disorders may add to limitations in functioning, e.g., problems to articulate or to eat as well as dysphagia [10, 11]. Sparing of the nervus facialis during surgery can help to maintain quality of life in certain domains [10], but of course, this is not always possible.

Emotional problems were sometimes mentioned as severe, especially anxiety, depression, and social isolation, whereas dissatisfaction with facial appearance was named only once in response to the open question. It is important to offer help to survivors with such problems, for example recommending that they make use of counseling or psychotherapy. There is good evidence that the course of health‐related quality of life is highly related to mental health [49, 50]. This calls for professional psychological support for patients with mental health problems, which is frequently insufficient [51, 52, 53], especially in men [54], even though head and neck cancer patients may have even higher levels of emotional problems than other cancer patients [55]. A systematic review and meta‐analysis has shown convincingly that different methods of psychotherapeutic interventions alleviate anxieties and depression in cancer patients and improve health‐related quality of life [56]. A recent randomized trial also showed that it is possible to increase participation in psychosocial counseling for men when they are addressed adequately [57].

All these findings should be interpreted in light of the study's limitations. First of all, the sample size is not very large, so the number of variables in our regression models was constrained. It is noteworthy that the differences between patients with unimodal treatment (surgery) and multimodal treatment (surgery combined with radiotherapy) were statistically significant despite this small sample in a number of quality‐of‐life domains. The reason for this might be that the differences in health‐related quality of life really are large. This is indeed relevant because the time since diagnosis was 10 years on average in our sample, which shows the long‐lasting effects multimodal treatment may have on the patient's quality of life, even though it is, of course, often necessary to prolong the patient's life.

Further, we were not able to document the current intake of medication of the survivors. As certain drugs can cause mouth dryness (e.g., anticholinergics), this could have confounded our findings if the survivors took such drugs more often than other people.

Another limitation is that we included only survivors who were able to participate in a clinical examination, which has likely introduced a healthy survivor bias to some extent. Moreover, we were not allowed to document data from survivors who declined participation in many institutions; hence we miss reliable information about potential selection bias. While our inclusion of an on‐site clinical examination ensures the results for the attending survivors are quite robust and valid, it could be that the survivors feeling less well were less likely to be able to participate. Therefore, our results may underestimate toxicities and quality of life impairment.

The cross‐sectional design prohibits concluding causation of our results, and we can only report associations. We also do not have exact information about the radiotherapy schedule the patients received. It is also not possible to investigate the course of quality of life or toxicity based on our data. One way of contextualizing the results is to compare them with published pre‐treatment findings, which we did with data from a clinical registry from South Korea [34]. Regarding the distribution of sex, education, and subsite, the Korean patients were comparable to the survivors in our study. However, they were about 10 years younger, which is plausible given that the diagnosis of our survivors dated back for about 10 years on average. Still, as age is often associated with quality of life, the comparisons must be done with caution.

To sum up, we investigated the quality of life and toxicities in salivary gland cancer survivors more than 5 years after diagnosis. Given the rarity of the condition, we were able to include a comparatively large sample of patients from different countries. We found that many survivors suffer from dry mouth, insomnia, trismus, sticky saliva, fatigue, and problems with sexuality, comparable with other head and neck cancer survivors. Soft tissue fibrosis, dry mouth, Frey's syndrome, and nerve disorders are the most common toxicities.

## Ethics Statement

All sites obtained ethical approval in accordance with regional and national requirements. Approval number from the principal investigator's institution: No. 2018‐13579.

## Consent

Patients were given time to consider the study and ask any questions before consenting and participating. All participants provided written informed consent.

## Conflicts of Interest

N.K. reports honoraria from ONO PHARMACEUTICAL, Bristol Meyers Squibb, Merck Biopharma, Astra‐Zeneca, Merck Sharp & Dohme, Merck Biopharma, Eisai, Bayer, Novartis and Chugai Pharmaceutical, all outside the submitted work. M.P. has received consulting fees from Meeting & Words S.r.l. and Hinovia S.r.l., and participation as Co‐investigator in a study funded by Amgen, all of which are outside of this study. S.S. reports honoraria for reviewing scientific papers from Lilly, outside the submitted work. The other authors declare no conflicts of interest.

## Supporting information


**Table S1:** Health‐related quality of life by type of radiotherapy.
**Table S2:** Health‐related quality of life by subsite.
**Table S3:** Health‐related quality of life by gender.
**Table S4:** Health care use.

## Data Availability

The data of this study are stored in the EORTC data repository and can be accessed by other researchers (https://www.eortc.org/data‐sharing/).
