# Supplementary material for: Late Toxicity and Long‐Term Quality of Life in Survivors of Cancer of the Major Salivary Glands More Than 5 Years After Diagnosis: A Multi‐National Study
Source: Head Neck. 2025 Aug 1;48(1):13–25. doi: 10.1002/hed.28263 (PMC12703571; doi:10.1002/hed.28263)
Supplement: Supplementary file 1 — Table S1: Health‐related quality of life by type of radiotherapy. Table S2: Health‐related quality of life by subsite. Table S3: Health‐related quality of life by gender. Table S4: Health care use. [file HED-48-13-s001.docx]

# Supplementary Material

## eTable 1 Health-related quality of life by type of radiotherapy

|  | Type of Radiotherapy | |  |
| --- | --- | --- | --- |
|  | **3D conformal** | **IMRT** | Delta |
| Number of survivors | **26** | **22** |  |
| Pain in the mouth | 16.3 | 13.3 | 3.1 |
| Swallowing | 9.6 | 6.1 | 3.6 |
| Dry mouth | 42.3 | 42.4 | -0.1 |
| Sticky saliva | 38.5 | 12.1 | 26.3 |
| Social eating | 9.7 | 4.9 | 4.8 |
| Social contact | 11.3 | 7.0 | 4.3 |
| Senses | 19.2 | 13.6 | 5.6 |
| Problems with teeth | 23.1 | 19.7 | 3.4 |
| Opening mouth | 30.8 | 25.8 | 5.0 |
| Sexuality | 26.7 | 34.1 | -7.5 |
|  |  |  |  |
| Emotional functioning | 78.5 | 81.3 | -2.8 |
| Global quality of life | 72.8 | 69.7 | 3.1 |

*Note: Displayed are the mean scores of quality of life domains. Higher scores indicate more symptom burden except for “Emotional functioning” and “Global quality of life”, where higher scores indicate better quality of life.*

## eTable 2 Health-related quality of life by subsite

|  | Subsite | |  |
| --- | --- | --- | --- |
|  | Parotid gland | Other salivary gland | Delta |
| Number of survivors | **47** | **13** |  |
| Pain in the mouth | 14.0 | 7.1 | 7.0 |
| Swallowing | 7.6 | 3.2 | 4.4 |
| Dry mouth | 39.0 | 30.8 | 8.2 |
| Sticky saliva | 22.0 | 28.2 | -6.2 |
| Social eating | 8.2 | 1.3 | 6.9 |
| Social contact | 9.1 | 5.6 | 3.4 |
| Senses | 17.0 | 5.1 | 11.9 |
| Problems with teeth | 24.1 | 2.6 | 21.5 |
| Opening mouth | 26.1 | 20.5 | 5.6 |
| Sexuality | 27.5 | 38.5 | -10.9 |
|  |  |  |  |
| Emotional functioning | 81.1 | 73.7 | 7.4 |
| Global quality of life | 72.0 | 65.4 | 6.6 |

*Note: Displayed are the mean scores of quality of life domains. Higher scores indicate more symptom burden except for “Emotional functioning” and “Global quality of life”, where higher scores indicate better quality of life.*

## eTable 3 Health-related quality of life by gender

|  | Gender | |  |
| --- | --- | --- | --- |
|  | **Men** | **Women** | Delta |
| Number of survivors | **31** | **29** |  |
| Pain in the mouth | 11.6 | 13.5 | -1.9 |
| Swallowing | 4.8 | 8.6 | -3.8 |
| Dry mouth | 30.1 | 44.8 | -14.7 |
| Sticky saliva | 20.4 | 26.4 | -6.0 |
| Social eating | 3.9 | 9.8 | -5.9 |
| Social contact | 8.6 | 8.0 | 0.6 |
| Senses | 14.5 | 14.4 | 0.1 |
| Problems with teeth | 17.2 | 21.8 | -4.6 |
| Opening mouth | 15.1 | 35.7 | -20.7 |
| Sexuality | 23.0 | 37.7 | -14.7 |
|  |  |  |  |
| Emotional functioning | 78.5 | 80.7 | -2.2 |
| Global quality of life | 68.8 | 72.4 | -3.6 |

*Note: Displayed are the mean scores of quality of life domains. Higher scores indicate more symptom burden except for “Emotional functioning” and “Global quality of life”, where higher scores indicate better quality of life.*

## eTable 4 Health care use

| At least one appointment during past 12 months with … | Number | Percentage |
| --- | --- | --- |
| Dentist | 54 | 90% |
| General practitioner | 48 | 80% |
| Head and neck surgeon, otolaryngologist | 22 | 37% |
| Cardiologist | 8 | 13% |
| Psychotherapist, psychologist, psychiatrist | 7 | 12% |
| Maxillo-facial surgeon | 5 | 8% |
| Oncologist | 5 | 8% |
| Neurologist | 5 | 8% |
| Radiation oncologist | 4 | 7% |
| Dietitian | 4 | 7% |
| Speech language pathologist, speech therapist | 3 | 5% |
